# Sepsis assessment in SJS/TEN: an important point overlooked? – Reply^☆^^☆☆^

**DOI:** 10.1016/j.abd.2019.06.003

**Published:** 2019-11-11

**Authors:** Carlos Gustavo Wambier, Sarah Perillo de Farias Wambier

**Affiliations:** a Department of Dermatology, Yale University School of Medicine, New Haven, United States; b Department of Medicine, Universidade Estadual de Ponta Grossa, Ponta Grossa, PR, Brazil

Dear Editor,

We were pleased to read the additional commentary on our article by Khurana et al.^1^ with the insight of adding procalcitonin as a serum marker for sepsis in severe epidermal necrolysis (EN) patients.

In many patients fever is attributed to the general inflammatory chaos of EN. Since prophylactic antibiotic therapy is not a standard routine and some patients have fever as an isolated infection signal, it seems reasonable to adopt a laboratory test to evaluate the likelihood of sepsis in patients with tachycardia, fever, or any other laboratory or clinical sign of infection not prompting antibiotic therapy. Thus, procalcitonin may be most helpful in the context of the initial expectant approach. A positive result should prompt change in intervention, and a negative result would be reassuring.

Hypothermia, on the other hand, could be a more specific clinical sign of sepsis. However, it is known to be associated with poor prognosis.^2^ Recently, procalcitonin > 1 μg/L and also hypothermia were associated with positive blood cultures in EN patients.^3^

Despite the presence of sepsis, therapeutic immunosuppression must not be delayed in patients with high SCORTEN. During the chart review^4^ some fatal cases were not prescribed systemic immunosuppression because of the possibility of sepsis, in a period of clinical decision preceding SCORTEN. Many of those patients could have had a different outcome if clinicians were aware that prediction of death was the most likely event according to SCORTEN, a game-changer in the treatment of EN.

## Financial support

None declared.

## Authors’ contribution

Carlos Gustavo Wambier: Approval of the final version of the manuscript; critical literature review; critical manuscript review; preparation and writing of the manuscript.

Sarah Perillo de Farias Wambier: Approval of the final version of the manuscript; critical literature review; critical manuscript review.

## Conflicts of interest

None declared.
